# Fournier's Gangrene: Current Practices

**DOI:** 10.5402/2012/942437

**Published:** 2012-12-03

**Authors:** M. N. Mallikarjuna, Abhishek Vijayakumar, Vijayraj S. Patil, B. S. Shivswamy

**Affiliations:** Department of General Surgery, Victoria Hospital, Bangalore Medical College and Research Institute, Bangalore 560002, India

## Abstract

Fournier's gangrene is an acute, rapidly progressive, and potentially fatal, infective necrotizing fasciitis affecting the external genitalia, perineal or perianal regions, which commonly affects men, but can also occur in women and children. There has been an increase in number of cases in recent times. Despite advanced management mortality is still high and averages 20–30%. Early diagnosis using Laboratory Risk Indicator for Necrotizing Fasciitis score and stratification of patients into high risk category using Fournier's Gangrene Severity Index score help in early initiation of treatment. Triple antibiotic combined with radical debridement is the mainstay of treatment. There have been many advances in management of Fournier gangrene including use of vaccum assisted closure and hyperbaric oxygen therapy. With introduction of newer devices like Flexi-Seal, fecal diversion can be done, avoiding colostomy. Reconstruction of perineal defects using skin grafts, flaps, and urethral reconstruction using gracilis flaps can reduce the morbidity associated with FG and provide acceptable functional and aesthetic outcomes.

## 1. Introduction 

**Figure 1:**
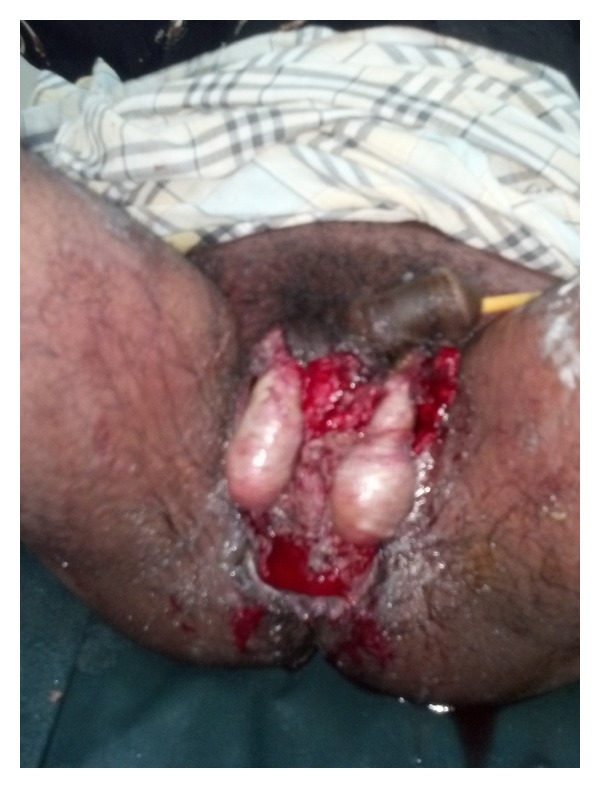
Partially debrided Fournier's gangrene.

Fournier's gangrene (FG) is an acute, rapidly progressive, and potentially fatal, infective necrotizing fasciitis affecting the external genitalia, perineal or perianal regions, which commonly affects men, but can also occur in women and children [1]. In 1764, Baurienne originally described an idiopathic, rapidly progressive soft-tissue necrotizing process that led to gangrene of the male genitalia. However, Jean-Alfred Fournier, a Parisian venereologist, is more commonly associated with this disease, which bears his name. In one of Fournier's clinical lectures in 1883, he presented a case of perineal gangrene in an otherwise healthy young man. Since Fournier's description, subsequent experience has shown that, in most cases, Fournier gangrene has an identifiable cause and that it frequently manifests more indolently. Over the years several terms have been applied to Fournier's gangrene including “streptococcus gangrene,” “necrotising fasciitis,” “periurethral phlegmon,” “phagedena,” and “synergistic necrotising cellulitis”. Early surgical debridement (as shown in Figure 1) of necrotic tissues and antibiotics are fundamental in the treatment of FG. Despite advanced management mortality is still high and averages 20–30% [2]. In a review of 1726 cases from 1950 to 1999 worldwide, reported in the English literature, the mortality rate was 16 per cent. In a subsequent unpublished study of 3297 cases of FG from 1950 to 2007, the mortality rate rose to 21.1%. This is in spite of advances in technology and medical practice. It was paradoxically observed in both studies that mortality was higher in the advanced countries of America, Canada, and Europe than in the underdeveloped countries [3].

## 2. Aetiology

Initially, FG was defined as an idiopathic entity, but diligent search will show the source of infection in the vast majority of cases, as either perineal and genital skin infections. Anorectal or urogenital and perineal traumas, including pelvic and perineal injury or pelvic interventions, are other causes of FG (Table 1). The most common foci include the gastrointestinal tract (30–50%), followed by the genitourinary tract (20–40%), and cutaneous injuries (20%) [3].

Comorbid systemic disorders are being identified more and more in patients with FG, the commonest being diabetes mellitus and alcohol misuse; other associations include extremes of age, malignancy, chronic steroid use, cytotoxic drugs, lymphoproliferative diseases, malnutrition, and HIV infection. Diabetes mellitus is reported to be present in 20–70% of patients with FG [4] and chronic alcoholism in 25–50% patients [5]. Any condition with decreased cellular immunity may predispose to the development of Fournier gangrene theoretically. The emergence of HIV into epidemic proportions has opened up a huge population at risk for developing FG [6]. 

## 3. Pathophysiology

Infection represents an imbalance between host immunity and the virulence of the causative microorganisms. The aetiologic factors allow the portal of entry of the microorganism into the perineum. The compromised immunity provides a favourable environment to initiate the infection, and the virulence of the microorganism promotes the rapid spread of the disease. Most authorities believe the polymicrobial nature of Fournier gangrene is necessary to create the synergy of enzyme production that promotes rapid multiplication and spread of the infection [7]. These organism are usual commensals of perineal skin and genital organs, and include Clostridia, Klebsiella, Streptococci, Coliforms, Staphylococci, Bacteriodes, and Corynebacteria [8]. Characteristically in Founier's gangrene exists synergism between theoretically low aggressive bacteria alone. For example, one microorganism might produce the enzymes necessary to cause coagulation of the nutrient vessels. Thrombosis of these nutrient vessels reduces local blood supply. Thus, tissue oxygen tension falls. The resultant tissue hypoxia allows growth of facultative anaerobes and microaerophilic organisms. These latter microorganisms, in turn, may produce enzymes (e.g., lecithinase, collagenase), which lead to digestion of fascial barriers, thus fueling the rapid extension of the infection [9]. The most commonly isolated aerobic microorganism are *Escherichia coli, Klebsiella pneumonia*, and *Staphylococcus aureus*. The most commonly isolated anaerobic microorganism is Bacteriodes fragilis [10]. Actually both aerobes and anaerobes are present in the tissues but anaerobes are less frequent isolated because these samples are more difficult to preserve. In some series, a mean of four different organisms is cultured from each patient [11]. Rare reports of other organisms being cultures include Candida albicans [12] and Lactobacillus gasseri [13]. 

Ultimately, an obliterative endarteritis develops, and the ensuing cutaneous and subcutaneous vascular necrosis leads to localized ischemia and further bacterial proliferation. Rates of fascial destruction as high as 2-3 cm/h have been described in some reports [14]. Infection of superficial perineal fascia (Colles fascia) may spread to the penis and scrotum via Buck and Dartos fascia, or to the anterior abdominal wall via Scarpa fascia, or vice versa. Perineal fascia is attached to the perineal body and urogenital diaphragm posteriorly and to the pubic rami laterally, thus limiting progression in these directions.

Deeper infection that extends below the facial layers to involve myonecrosis isnot generally thought to be a feature of classical Fournier's gangrene, although it has been described [15]. Testicular involvement is rare in Fournier's gangrene because of the separate blood supply to the testes [16]. Ayan et al. [17] reviewed 41 cases of Fournier's gangrene and found that a bilateral orchidectomy was performed in 4 patients and a unilateral orchidectomy was performed in 5 patients. In his large review of 1726 patients Eke [3] suggested that when testicular involvement does occur it indicates a retroperitoneal or intra-abdominal source of infection. Penis involvement is also rare and the corpora are usually spared while the skin sloughs off. Thrombosis of the corpus spongiosum and cavernosum has, however, been reported [18]. 

## 4. Clinical Features

The clinical features of Fournier's gangrene include sudden pain in the scrotum, prostration, pallor, and pyrexia. At first only the scrotum is involved, but if unchecked, the cellulitis spreads until the entire scrotal coverings slough, leaving the testes exposed but healthy [19]. The presentation may also be insidious as opposed to the classical sudden onset presentation. One overwhelming feature of the presentation is the strong “repulsive, fetid odour” that is associated with the condition [20]. Patients can present with varying signs and symptoms including fever greater than 38°C, scrotal swelling and erythema, purulence or wound discharge, crepitation, or fluctulance [21]. In their case series Ferreira et al. [22] found that the most common presentations were scrotal swelling, fever, and pain. The mean interval between initial symptoms and arrival at the hospital was 5.1 ± 3.1 days. Scrotal involvement was found in 93.3% of cases, the penis was involved in 46.5% of cases, and the perineum or perianal region was involved in 37.2% of cases. Ersay et al. [23] found that the most common presentation was perianal/scrotal pain (78.6%) followed by tachycardia (61.4%), purulent discharge from the perineum (60%), crepitus (54.3%), and fever (41.4%). Crepitus of the inflamed tissue is a common feature of the disease due to the presence of gas forming organisms. As the subcutaneous inflammation worsens, necrotic patches start appearing over the overlying skin and progress to extensive necrosis [24]. The spread of infection is along the facial planes and is usually limited by the attachment of the Colles' fascia in the perineum [25]. Infection can spread to involve the scrotum, penis, and can spread up the anterior abdominal wall, up to the clavicle [26]. 

## 5. Investigations

Although the diagnosis of Fournier gangrene is most commonly made clinically, laboratory studies are required in early setting and also for risk stratification and prediction of mortality. Many imaging modalities are also used to diagnose Fournier's gangrene and also to find out the etiology.

## 6. Laboratory Studies

The following studies are indicated in patients Fournier gangrene.

- CBC with differential count.
- Electrolytes, BUN, creatinine, blood glucose levels: acidosis with hyperglycemia or hypoglycemia may be present. Dehydration occurs as the disease progresses.
- ABG sampling to provide a more accurate assessment of acid/base disturbance.
- Blood and urine cultures.
- Disseminated intravascular coagulation (DIC) panel (coagulation studies, fibrinogen/fibrin degradation product levels) to find evidence of severe sepsis.
- Cultures of any open wound or abscess.

The Laboratory Risk Indicator for Necrotizing Fasciitis (LRINEC) is a robust laboratory measurement score capable of determining even clinically early cases of necrotizing fasciitis (Table 2) [27]. Using logistic regression analysis of independent variables from 89 cases of necrotizing fasciitis factors were identified to be independent predictors. Of the cohort of 89 patients only 13 (14.6%) patients had a diagnosis or suspicion of necrotizing fasciitis on admission. A majority were therefore missed, resulting in delayed operative debridement. In contrast, 80 (89.9%) of these patients had a LRINEC score of ≥6. According to Wong et al. the biochemical and hematologic changes in necrotizing fasciitis develop early in the evolution of the disease and the LRINEC score can stratify patients into high and moderate risk categories even when the clinical picture is still equivocal. A LRINEC score of ≥6 should raise the suspicion of necrotizing fasciitis among patients with severe soft tissue infections, and a score ≥8 is strongly predictive of this disease.

In a large retrospective study of 68 patients, Corcoran et al. [28] described significant differences between nonsurvivors and survivals in admission laboratory parameters such as high serum creatinine, lactate, and calcium or low bicarbonate. Increased calcium in serum may be due to renal failure, bacteriemia, or use of parenteral nutrition. A study by Erol et al. [29] demonstrated that admission hypomagnesaemia is associated with high mortality in critical ill patients. Reduced intestinal absorption, increased urinary losses or intracellular shift are possible reasons of this action. Monitoring serum magnesium levels in patients with Fournier's Gangrene might have prognostic and therapeutic implications and is used today in specialized groups.

In a study published by Laor et al. [30] for the first time a Fournier's gangrene severity index (FGSI) along the lines of the Acute Physiology and Chronic Health Evaluation score (APACHE II) was described. They identified several prognostic factors associated with a worse prognosis. In the FGSI score, nine parameters were calculated: temperature, heart rate, respiratory rate, serum sodium, potassium, creatinine, bicarbonate levels, hematocrit, and leukocyte. The degree of derivation from normal is graded from 0 to 4. The individual values are summed to obtain the FGSI score. Results published in the articles shows that a score >9 has 75% of death and patients with a score <9 were associated with 78% of survival. Other series of patients analyzed with the same score shows FGSI >10.5 is associated with 96% of death and <10.5 96% of survival [31]. Yilmazlar et al. made modification to FGSI with increased prognostic indication (Table 3) [32].

## 7. Imaging Studies of Fournier Gangrene

### 7.1. Conventional Radiography

At radiography, hyperlucencies representing soft tissue gas may be seen in the region overlying the scrotum or perineum. Subcutaneous emphysema may be seen extending from the scrotum and perineum to the inguinal regions, anterior abdominal wall, and thighs. However, the absence of subcutaneous air in the scrotum or perineum does not exclude the diagnosis of Fournier gangrene. Up to 90% of patients with Fournier gangrene have been reported to have subcutaneous emphysema, so that at least 10% do not demonstrate this finding [33]. Radiography may also demonstrate significant swelling of scrotal soft tissue. Deep fascial gas is rarely seen at radiography, which represents a significant weakness of this modality in the diagnosis and evaluation of Fournier gangrene [34].

### 7.2. Ultrasonography

A US finding in Fournier gangrene is a thickened wall containing hyperechoic foci that demonstrate reverberation artifacts, causing “dirty” shadowing that represents gas within the scrotal wall. Evidence of gas within the scrotal wall may be seen prior to clinical crepitus. Reactive unilateral or bilateral hydroceles may also be present. If testicular involvement occurs, there is likely an intraabdominal or retroperitoneal source of infection. US is also useful in differentiating Fournier gangrene from inguinoscrotal incarcerated hernia; in the latter condition, gas is observed in the obstructed bowel lumen, away from the scrotal wall [35]. 

### 7.3. Computed Tomography

The CT features of Fournier gangrene include soft-tissue thickening and inflammation. CT can demonstrate asymmetric fascial thickening, any coexisting fluid collection or abscess, fat stranding around the involved structures, and subcutaneous emphysema secondary to gas-forming bacteria. The underlying cause of the Fournier gangrene, such as a perianal abscess, a fistulous tract, or an intraabdominal or retroperitoneal infectious process, may also be demonstrated at CT. In early Fournier gangrene, CT can depict progressive soft-tissue infiltration, possibly with no evidence of subcutaneous emphysema. Because the infection progresses rapidly, the early stage with lack of subcutaneous emphysema is brief and is rarely seen at CT [36]. 

## 8. Treatment and Management

The cornerstones of treatment of Fournier's gangrene are urgent surgical debridement of all necrotic tissue as well as high doses of broad-spectrum antibiotics. Urgent resuscitation with fluids as well as blood transfusions may be needed and use of albumin and vasopressors in patients who present with shock to improve hemodynamics may be also needed.

### 8.1. Broad Spectrum Antibiotics Coverage

Empiric broad-spectrum antibiotic therapy should be instituted as soon as possible, until the culture results could make adjusted the therapy. The antibiotic regimen chosen must have a high degree of effectiveness against staphylococcal and streptococcal bacteria, gram-negative, coliforms, pseudomonas, bacteroides, and clostridium. Classically Triple therapy is usually recommended. Third generation cefalosporins or aminoglycosides, plus penicillin and metronidazole. Clindamycin may be used as it is shown to suppress toxin production and modulate cytokine production; also use of linezolide, daptomycine, and tigecycline is warranted in cases of previous hospitalizations with prolonged antibiotic therapy which may lead to resistant bacteroides [37]. New clinical guidelines currently recommend the use of Carbapenems (Imipenem, meropenem, ertapenem) or piperaziline-tazobactam. These newer drugs have larger distribution and lesser renal toxicity in comparison to aminoglycosides. This new trend suggests that classically triple therapy could be replaced in certain circumstances for the use of new generation antibiotics [38]. 

### 8.2. Radical Surgical Debridement

A debridement of the necrotic tissue as soon as possible it is widely recommended Laor et al. found no significant difference between the onset time of symptoms, early surgical treatment and mortality, but other studies from Kabay et al. [31] and Korkut et al. [39] show that this time interval should be as short as possible. Debridement of deep fascia and muscle is not usually required as these areas are rarely involved similar to testes. Debridement should be stopped when separation of the skin and the subcutaneous is not perform easily, because the cutaneous necrosis is not a good marker. Multiple surgical debridement is the rule rather than the exception, with an average of 3.5 procedures required per patient [40]. In some series orchiectomy was performed because of observed severe infection in peritesticular tissues, although in the pathological review the testicles were not found to be involved [8]. It is possible to temporally place the testes into subcutaneous pouch until healing or reconstruction complete [1]. 

### 8.3. Fecal and Urinary Diversion

 Colostomy has been used for fecal diversion in cases of severe perineal involvement. The rationale for rectal diversion includes a decrease in the number of germs in perineal region and improved wound healing. Justifications for its construction are anal sphincter involving, fecal incontinence, or continues fecal contamination of the wound's margins. In several papers, the percentage of patients with a colostomy is around 15% depending on the series [8]. In contrast to the study of Corcoran et al. [28], other series reported that formation of a diverting colostomy was associated with increased mortality [29]. Korkut et al. reported 45 cases of FG and showed that mortality among patients not requiring a stoma was 7%, but was 38% among patients in whom stoma was required. Diverting colostomy does not eliminate the necessity of multiple debridements, nor reduces the number of these procedures. However this associated technique may lead to early oral intake and thus may help to improve the wound cure process with better nutrition and less contamination of wounds. Anyway, serious stoma-related complications were described like wound infection, stomal ischemia, and evisceration [41].

**Figure 2:**
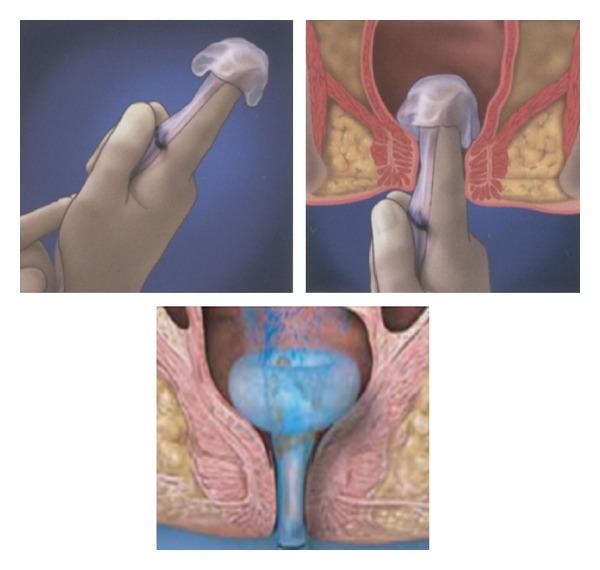
Flexi-Seal Fecal Management system.

 Rectal diversion device The Flexi-weal Fecal Management system (Figure 2) is a silicone catheter designed to divert fecal matter in patients with diarrhea, local burns, or skin ulcers. The device protects the wounds from fecal contamination and reduces the same way that a colostomy both the risk of skin breakdown and repeated inoculation with colonic flora. Estrada et al. [42] showed that it was effective way for fecal diversion and forms an alternative to colostomy. It is recommended to explore the canal anal before placement of the catheter in order to avoid rectal injuries. This device avoids complications related to stomas, including better psychological recovery of the patient and also may have an economic benefit. A formal contraindication is rectal neoplasm, penetrating rectal injuries or fistulas. Urinary diversion becomes necessary when there is penile or urethral involvement though some cases may require cystostomy, urinary catheterization may suffice in many.

### 8.4. Topical Therapy

There have been reports of use of honey to aid wound healing. Honey has a low pH of 3.6 and contains enzymes which digest necrotic tissues it also has antibacterial property due to phenolic acid. These changes occurs within a week of applying honey to the wound. Unfortunately there is no randomized study about the efficacy of honey in this special situations. Heggers et al. [43] showed that irrigation of wounds with sodium hypochlorite to a concentration of 0.025% will safely and effectively treat FG. Additionally, this concentration had been proven to be not only bactericidal but also nontoxic to host tissues. In a study by Altunoluk et al. [44] it was shown that Dakin solution dressing reduced hospital stay morbidity and mortality. Application is justified when hydrogen peroxide is used in the correct circumstances, but precautions should be taken when used in closed spaces or under pressure, where liberated oxygen cannot escape, and dangerous side effects are described as blood oxygen embolism. From the same way, hydrogen peroxide subcutaneous crepitus can be confused with typical disease progression [45]. 

Enzymatic debridements with lyophilized collagenase application are other local treatment that have been shown to be beneficial [46]. Use of fibrin glue has recently been suggested in skin defects with no active infection [47]. 

### 8.5. Hyperbaric Oxygen Therapy

Hyperbaric oxygen therapy implies placing the patient in an environment of increased ambient pressure while breathing 100% oxygen, resulting in enhanced oxygenation of the arterial blood and tissues and demonstrated benefits of hyperbaric oxygen include adequate oxygenation for optimal neutrophil phagocytic function, inhibition of anaerobic growth, increased fibroblast proliferation and angiogenesis, reduction of edema by vasoconstriction, and increased intracellular antibiotics transportation [48]. Hypoxia may also reduce the effectiveness of several antibiotics (vancomycin, ciprofloxacin) while hyperoxia may help others. For example aminoglycosides cross the cell membrane of the microorganism by an oxygen-dependent pump. In addition, some side effects have been described as toxic reaction of central nervous system and barotrauma injury to the middle ear. The use of hyberparic oxygen therapy continues to be cause for debate. Certainly no prospective controlled trials have been published for this condition. Although this treatment is supported by some small studies, hyperbaric oxygen should not delay surgical debridement [49]. 

### 8.6. Vacuum-Assisted Closure

With the recent advent of the vacuum assisted closure (VAC) system dressing, there seems to be a dramatic improvement with minimising skin defects and speeding tissue healing. It simply works by exposing a wound to subatmospheric pressure for an extended period to promote debridement and healing. Weinfeld et al. treated four consecutive cases using negative pressure dressings (VAC) to bolster skin grafts in male genital reconstruction. In this series reconstruction followed one case of tumour ablation and three cases of debridement of abscesses or FG. The VAC was applied circumferentially to the penis to secure skin grafts either directly to the penile shaft or to facilitate skin grafting to the scrotum. Graft areas ranged from 75 cm to 250 cm. All cases resulted in successful genital wound coverage; minor complications are described [50]. 

### 8.7. Plastic Reconstruction

Various workers have used different techniques to provide skin cover including transplantation of testes, free skin grafts, axial groin flaps, and myocutaneous flaps. Split thickness skin graft seems to be the treatment of choice in treating perineal and scrotal skin defects. Parkash and Gajendran [51] reported their series of treatment of 43 cases in the past 11 years. In three cases the gangrene had spread beyond the scrotum and penis and cover had to be supplemented with split-skin grafts. In all the other cases, cover was provided with scrotal skin remnants at the edge of the lesion and on the penis with the inner layer of the prepuce, which had remained intact. 

On the other hand Black et al. [52] reported their series of meshed unexpanded split-thickness skin grafting (STSGs) for skin defects. They treated nine patients with penile skin loss between March 2001 and January 2003, with meshed STSGs to the penis. The underlying condition was FG in four cases, chronic lymphoedema in two, skin deficiency from previous surgeries in two, and Crohn's disease in one. Graft thickness was 0.012 or 0.016 inches and meshing was performed in a 1 : 1 ratio. Meshed slits were oriented transversely without expansion and the graft juncture was located on the ventral surface in zigzag fashion. Graft take, appearance, and sexual and voiding function were assessed postoperatively. All nine patients had 100% graft take. At a mean followup of six months a satisfactory cosmetic outcome was reported photographically in all except one case involving chronic penile manipulation. Erectile function and ejaculation were preserved in potent patients. 

Myocutaneous flap as Gracilis muscle flap is useful in some cases. Patients with a large and deep perineal defect often need this technique to eliminate the dead space. The well vascularized muscle flap demonstrates greater resistance to bacterial inoculums and in wounds with some degree of contamination [53]. Another alternative is the pudendal thigh flap. It is a fasciocutaneous flap based on the terminal branches of the superficial perineal artery, which arises from the internal pudendal artery. The advantages of this flap are relative simplicity and good blood supply. The donor site can be closed primarily and no muscle function is sacrificed.

Urethral reconstruction through a variety of methods including anterolateral thigh flaps, radial artery forearm free flap, and other simple skin and mucosal flaps were being done. Recently the use of prefabricated gracilis myocutaneous flap for long segment urethral reconstruction has been advocated [54]. 

## Figures and Tables

**Table 1:** Etiology of Fournier's gangrene.

*Anorectal *
Trauma
Ischiorectal, perirectal, or perianal abscesses, appendicitis,
diverticulitis, colonic perforations
Perianal fistulotomy, perianal biopsy, rectal biopsy,
hemorrhoidectomy, anal fissures excision
Steroid enemas for radiation proctitis
Rectal cancer
*Genitourinary *
Trauma
Urethral strictures with urinary extravasation
Urethral catheterization or instrumentation,
penile implantsinsertion, prostatic biopsy, vasectomy,
hydrocele aspiration,genital piercing, intracavernosal cocaine
injection Periurethral infection; chronic urinary tract infections
Epididymitis or orchitis
Penile artificial implant, foreign body
Hemipelvectomy
Cancer invasion to external genitalia
Septic abortion
Bartholin's duct abscess
Episiotomy
*Dermatologic sources *
Scrotal furuncle
Genital toilet (scrotum)
Blunt perineal trauma; intramuscular injections, genital piercings
Perineal or pelvic surgery/inguinal herniography.
*Idiopathic *

**Table 2:** Laboratory Risk Indicator for Necrotizing Fasciitis (LRINEC) score.

Variable, Units	Score
C-Reactive protein, mg/L	
<150	0
≥150	4
Total white cell count, per mm^3^	
<15	0
15–25	1
>25	2
Haemoglobin, g/dL	
>13.5	0
11–13.5	1
<11	2
Sodium, mmol/L	
≥135	0
<135	2
Creatanine, *μ*mol/L	
≤141	0
>141	2
Glucose, mmol/L	
≤10	0
>10	1

**Table 3:** The Uludag Fournier's gangrene severity index.

Physiological variable					Normal				
	+4	+3	+2	+1	0	+1	+2	+3	+4
Temperature (C)	>41	>39		38.5– 38.9	36–38.4	34– 35.9	32– 33.9	<31.9	<29.9
Heart rate	>180	140– 179	110– 139		70–109		55– 69	40–54	<39
Respiratory rate	>50	35– 49		25–34	12–24	10- 11	6–9		<5
Na^+^ (mmol/L)	>180	160– 179	155– 159	150– 154	130–149		120– 129	111– 119	<110
K^+^ (mmol/L)	>7	6–6.9		5.5– 5.9	3.5–5.4	3–3.4	2.5– 2.9		<2.5
Creatinine (mg/dL)	>3.5	2–3.4	1.5– 1.9		0.6–1.4		<0.6		
Hematocrit (%)	>60		50– 59.9	46– 49.9	30–45.9		20– 29.9		<20
Leucocytes (×10^3^/mm^3^)	>40		20– 39.9	15– 19.9	3–14.9		1–2.9		<1
Bicarbonate (mmol/L)	>52	41– 51.9		32– 40.9	22–31.9		18– 21.9	15– 17.9	<15

*Dissemination score *									
Fournier's gangrene confined to the urogenital and/or anorectal region, add 1									
Fournier's gangrene confined to the pelvic region, add 2									
Fournier's gangrene extending beyond the pelvic region, add 6									

*Age score *									
Age ≥ 60 years, add 1									
